# Electrochemical exfoliation of graphene from pencil lead

**DOI:** 10.1038/s41598-024-66825-0

**Published:** 2024-07-10

**Authors:** Natchanon Jiwarawat, Thapan Leukulwatanachai, Kunbhass Subhakornphichan, Siwagorn Limwathanagura, Sittinadh Wanotayan, Nithi Atthi, Apirak Pankiew, Porpin Pungetmongkol

**Affiliations:** 1 International School of Engineering, Nano-Engineering, Faculty of Engineering, Chulalongkorn University, Bangkok, 10330 Thailand; 2 Department of Chemical Engineering, Bio-Circular-Green-economy Technology & Engineering Center, BCGeTEC, Chulalongkorn University, Bangkok, 10330 Thailand; 3 Thai Microelectronics Center (TMEC), National Science and Technology Development Agency (NSTDA), 111 Thailand Science Park, Phahonyothin Road, Khlong Nueng, Khlong Luang, 12120 Pathum Thani Thailand

**Keywords:** Chemistry, Engineering, Materials science, Nanoscience and technology, Physics

## Abstract

Addressing an ever-increasing demand for graphene in recent years, simple, accessible, and effective graphene synthesis methods are essential. One of such methods is to use a highly oriented pyrolytic graphite (HOPG) to perform an electrochemical exfoliation. While this is one of the simplest and most cost-effective methods, the limited availability and price of HOPG hinders its usage. Our study proposed a simple and economical electrochemical exfoliation of pencil lead, producing graphene with properties comparable to that produced from HOPG. The electrical properties are determined by depositing graphene onto a screen-printed electrode. Graphene from pencil leads can achieve an electrical resistance as low as 1.86 kΩ, marking over 80% improvement in electrical performance compared to bare electrodes. This finding provides an alternative for the synthesis of graphene, increasing its availability and the cost-effectiveness as well as contributing towards a potential commercialization of the method in the future.

## Introduction

Graphene is a single atomic plane of carbon arranged in a hexagonal network^1^. Since its successful isolation by Andre Geim and Konstantin Novoselov in 2004^2^, graphene has been extensively studied for its exceptional properties, such as electrical conductivity, optical transparency, flexibility, and high mechanical strength^1^. Multiple methods can be used to synthesize graphene, each with its advantages, disadvantages, and target applications^3^. For instance, mechanical exfoliation is inexpensive to obtain high-quality graphene; however, this method still provides a relatively low yield^4^. Many graphene chemical exfoliation synthesis methods, adopted from the well-known Hummer’s method, have been utilized for many decades; however, they are limited to the production of graphene oxide, which requires a reduction process and involves handling various toxic chemicals and gases^5,6^. Other advanced methods, such as chemical vapor deposition (CVD) and epitaxial growth, result in high-quality graphene while being reliable and scalable but require specialized instruments to provide and sustain a high-temperature environment^7,8^. One of the more accessible and practical methods is electrochemical exfoliation due to its simplicity and lack of requirement for advanced instruments^9,10^. On the other hand, an electrochemical exfoliation method produces a lower-quality graphene, containing defects from the oxygen atoms in the non-reduced graphene oxide. A commonly used graphite precursor is a graphite rod/film/HOPG^11^. In recent years. there have been several attempts to synthesize graphene by using electrochemical exfoliation of alternative graphite sources such as graphite pencil leads^12–16^. However, the resulting graphene was still a mixture of graphene and highly defective graphene oxide for both precursors.

**Figure 1:**
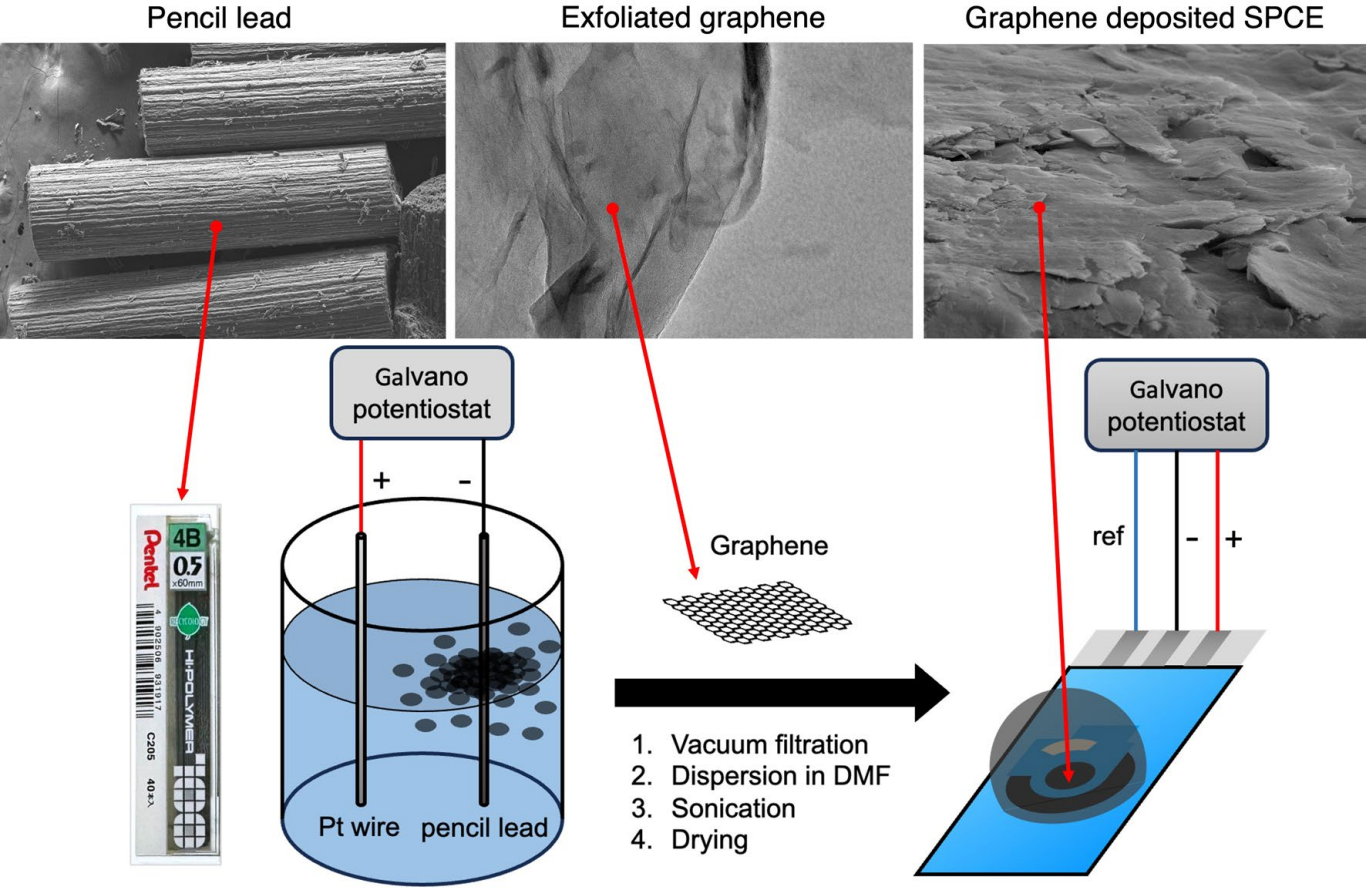
(top) FESEM image of the pencil lead, TEM image of exfoliated graphene, and cross-sectional FESEM image of graphene deposited on SPCE. (bottom) Overview of the graphene synthesis process using a pencil lead electrode and the graphene deposition process onto the SPCE and SPGE.

The objective of this study is to apply the electrochemical exfoliation process to demonstrate the potential of pencil lead to produce high-quality graphene with high conductivity and low defects without using the less ubiquitous HOPG. Through a controlled electrochemical process, vacuum filtration, and surfactant sonification, the high-quality graphene product can be obtained. The electrical property of graphene was validated by depositing the exfoliated graphene on the screen-printed carbon electrode (SPCE) and screen-printed graphene electrode (SPGE). The design requirement will focus on both quantitative and qualitative metrics. The quantitative metrics include the percentage of carbon and oxygen atoms in the material, the I_D_/I_G_ ratio which indicates the amount of defect present in the structure, high-resolution XPS spectra of C 1s analysis and the conductivity of the SPCE and SPGE deposited with graphene. The qualitative metrics include the overall morphological, crystallographic structure and characteristic Raman spectrum of the synthesized graphene. Figure 1 provides an overview of the entire process of graphene synthesis and its deposition on the electrode.

## Results and discussions

### Exfoliated graphene from pencil lead vs HOPG source

By varying the alternating potentials for different periods of time (2, 5, and 10 s), alternating between positive and negative potential (2/2, 5/5, and 10/10), it was observed that the appearance of the solution containing 5/5 and 10/10 Pencil Lead exhibited same results, displaying a clear black colloid in Supplementary Fig. S1. Meanwhile, the solution containing 2/2 Pencil Lead is transparent, indicating that it contains a negligible amount of graphene product. Thus, at least 5 s for each alternating potential is required to exfoliate a graphene sheet.

The EDS analysis of the graphene sources (pencil lead and HOPG) and graphene exfoliated powder from both sources was compared in Supplementary Table S1. The result indicated that HOPG was composed of 94.3% carbon with small percentage of oxygen. Whereas, the pencil lead has 88.9% carbon, 5.4% oxygen and other elements such as phosphorus, silicon and tin, which potentially resulted from the clay and impurities in pencil lead. The 10/10 HOPG has the highest percentage of carbon at 70.2, followed by the 5/5 HOPG, 5/5 Pencil Lead, and 10/10 Pencil Lead at 68.4, 60.4, and 58, respectively. Other elements as sulfur and potassium are also found in both products which could be from the electrolytes during the synthesis process. Graphene’s HOPG products demonstrated relatively higher carbon percent and lower oxygen percent due to its high purity HOPG source, nevertheless graphene from pencil lead could exhibit slightly different from its costly counterpart, HOPG.

**Figure 2:**
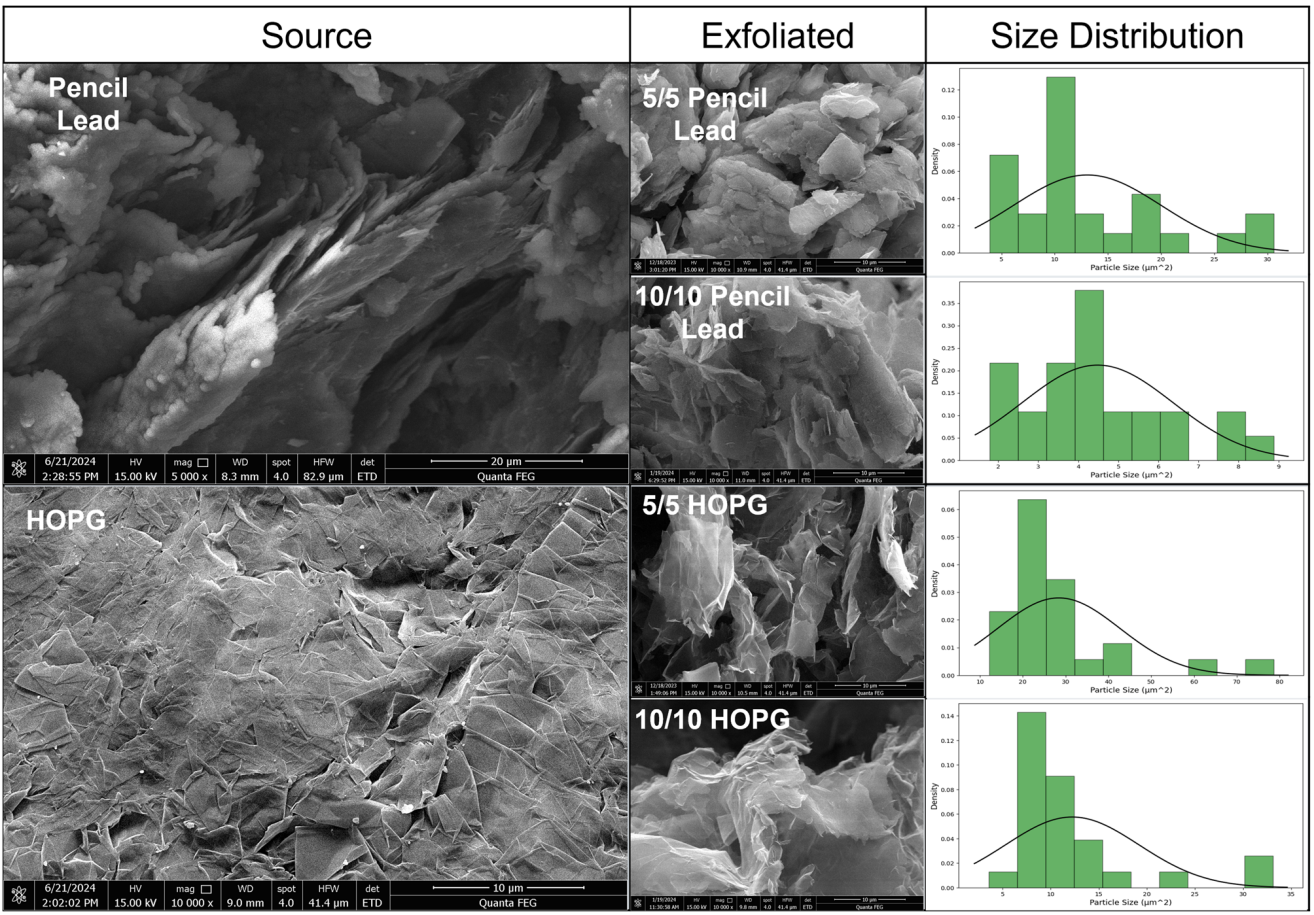
FESEM images of source materials: Pencil lead and HOPG and their exfoliated graphene products under different conditions (5/5 Pencil Lead, 10/10 Pencil Lead, 5/5 HOPG, and 10/10 HOPG). (Right) Size distribution of exfoliated graphene. The SEM images were measured using ImageJ version 1.54 g and the size distribution was plotted using Python 3.

FESEM images of source materials HOPG and pencil lead, and their exfoliated powders (5/5 Pencil Lead, 10/10 Pencil Lead, 5/5 HOPG, and 10/10 HOPG) are illustrated in Fig. 2. The HOPG source has very smooth and flat surface whereas pencil lead showed stacking layer of stiff graphite. Graphene exfoliated from HOPG exhibits characteristics of thin and flexible large sheets with some folded edges, while the graphene exfoliated from pencil lead was noticeably thicker and more rigid. This difference might be attributed to the purer composition of the graphitic sheet in HOPG with smooth surface as characteristic of its source opposing to pencil lead which showed stiff flake.

The result of size distribution was obtained from image analysis of each sample using ImageJ software. The average size of pencil lead of 5/5 and 10/10 powder are 13.067 and 4.481 µm^2^, respectively. Meanwhile, the average size of HOPG of 5/5 and 10/10 are 28.417 and 12.240 µm^2^, respectively. The 5/5 Pencil Lead was a lot larger than 10/10 product which might be due to the longer duration of exfoliation leading to more damage on graphene particles. The size of graphene exfoliated from HOPG with 5/5 condition compared with 10/10 was consistent with that graphene from pencil lead in which the 5/5 Pencil Lead yields larger sizes on average. As a results, both graphene exfoliated from pencil lead presents smaller particulate structure compared to large fabric like layer of graphene’s HOPG. Smaller size of graphene particles produced from pencil lead was probably due to the stiffness of the graphite flake obtained from pencil lead source.

The stiffness analysis of graphene exfoliated from HOPG and pencil lead have been tested and confirmed using AFM nanoindentation^17^. The information of nanoindentation stiffness test was illustrated in Supplementary Fig. S2 which also presented the force-separation curves of the graphene deposition on array of 2 µm diameter microwells silicon wafer. The schematic, AFM, and optical microscopic images of array micron wells fabricated on Silicon wafer were visualized in the Supplementary Fig. S2. The contact stiffness were obtained from the slope of the unloading curve using linear fit function in OriginLab based on the results collected from AFM nanoindentation. The pencil lead graphene yields higher stiffness than those exfoliated from HOPG. The stiffness of 5/5 and 10/10 graphene exfoliated from pencil leads are 53.52 and 71.21 N/m respectively. On the other hand, the stiffness of graphene exfoliated from HOPG with 5/5 and 10/10 are 20.19 and 27.15 N/m. The higher stiffness of the graphene exfoliated from pencil lead samples contributes to the formation of smaller graphene particles in both the 5/5 and 10/10 samples after the exfoliation process. While the HOPG has large sheet-like flake which reduce the stiffness of its overall structure. The film thickness and roughness were also assessed using AFM characterization. In the 5/5 condition, the film thickness of the 5/5 Pencil Lead is 186 nm with a roughness of 62 nm, while the 5/5 HOPG has a thickness of 198 nm and a roughness of 64 nm. In contrast, the 10/10 condition yielded the following results: the 10/10 Pencil Lead has a film thickness of 207 nm with a roughness of 176 nm, and the 10/10 HOPG has a thickness of 375 nm and a roughness of 167 nm. Overall, the graphene produced from HOPG source has a thicker film compared to those from pencil lead, and the 5/5 condition has produced smoother surface compared to the 10/10 when deposited.

**Figure 3:**
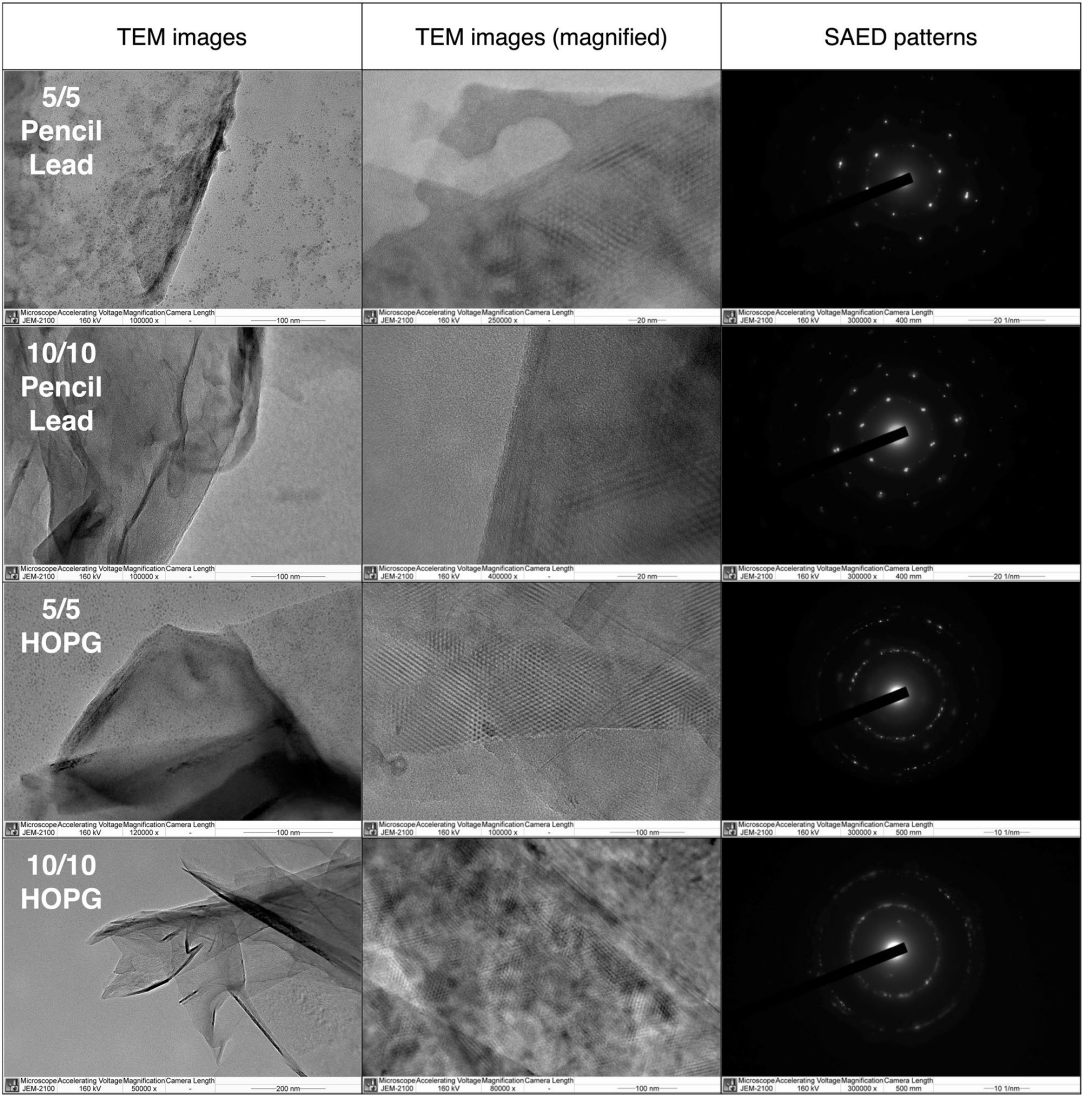
TEM images and SAED patterns of 5/5 Pencil Lead, 10/10 Pencil Lead, 5/5 HOPG and 10/10 HOPG.

Figure 3 shows the TEM images and the selected-area electron diffraction (SAED) pattern of 5/5 Pencil Lead, 10/10 Pencil Lead, 5/5 HOPG, and 10/10 HOPG. From the TEM images, graphene sheets can be clearly observed, confirming that graphene can be successfully synthesized in all four conditions. Second column of TEM images also show the distinctive hexagonal moiré pattern present in all four samples, which further verify the presence of graphene. From the SAED patterns, both 5/5 Pencil Lead and 10/10 Pencil Lead produce sharp dots arranged in a hexagonal pattern, indicating that highly monocrystalline graphene can be produced by the pencil lead electrodes. On the other hand, both 5/5 HOPG and 10/10 HOPG produce graphene with a polycrystalline structure as indicated by the less sharp dots forming circular rings. The blurry dots can be observed from the 10/10 HOPG, suggesting the highest deviation from the monocrystalline structure.

The Raman spectra of all samples revealed the presence of characteristic graphene peaks, including the D peak (1350 cm^−1^), G peak (1580 cm^−1^), and 2D peak (2685 cm^−1^) presented in Supplementary Fig. S3. The low I_D_/I_G_ ratio across all conditions implies the production of high-quality graphene with minimal defects, averaging around 0.2. This suggests the synthesis of graphene, rather than graphene oxide in agreed with low oxygen percentage in EDS result. Notably, the I_D_/I_G_ ratio is relatively smaller for pencil lead products, indicating fewer defects compared to HOPG graphene which was agreed with SAED result. In addition, this observation is consistent with FESEM results and stiffness tests, suggesting that the stiffer and smaller graphitic sheets of pencil lead are more resistant to damage during the exfoliation process compared to the flexible graphene sheet of HOPG, resulting in less defect product.

Number of layers of graphene can be confirmed from the I_2D_/I_G_ ratio from Raman spectroscopy. The high I_2D_/I_G_ ratio represents the low number of layers of graphene; however, the graphene samples in this study resulted in the intensity of G peak exceed that of the 2D peak as shown in Supplementary Fig. S3. This was attributed to the high analysis volume of the Raman spectroscopy, consequently averaging the response from the single layer to multiple layers graphene which were all presented in the exfoliated samples. Despite this, the 2D peak exhibited a relatively narrow profile, indicating a non-overlap of multiple peaks, which could infer that single-layer graphene was obtained from the synthesis process. 2D peak position and peak overlap of Raman spectra are considered to be a suitable method to identify the number of layers of graphene obtain from top-down approach as electrochemical exfoliation as in our case^18^. The 2D Raman spectral peak of powder obtained from HOPG and pencil leads were presented in Supplementary Fig. S3. From the figure, the 2D peak of 10/10 Pencil Lead was observed at the lowest wavenumber of 2682.42 cm^−1^, corresponding to graphene with less than 5 layers. 5/5 Pencil Lead also exhibited the peak at 2691.64 cm^−1^, indicating that graphene with less than 8 layers was obtained. This difference could originate from the increased in the duration of applied potential during the exfoliation process. As the potential was applied for a more prolonged duration, the graphite can exfoliate more effectively, consequently forming a lower number of graphene layers. For 5/5 HOPG and 10/10 HOPG, the 2D peak was observed at 2700.85 and 2710.04 cm^−1^, respectively. These peaks were already in the range of graphite, implying that multi-layer graphene was obtained from both conditions. The result of multiple layers graphene product could be caused by the flexible structure of graphene HOPG which could easily fold and stack, resulting in greater number of layers graphene product in average.

**Figure 4:**
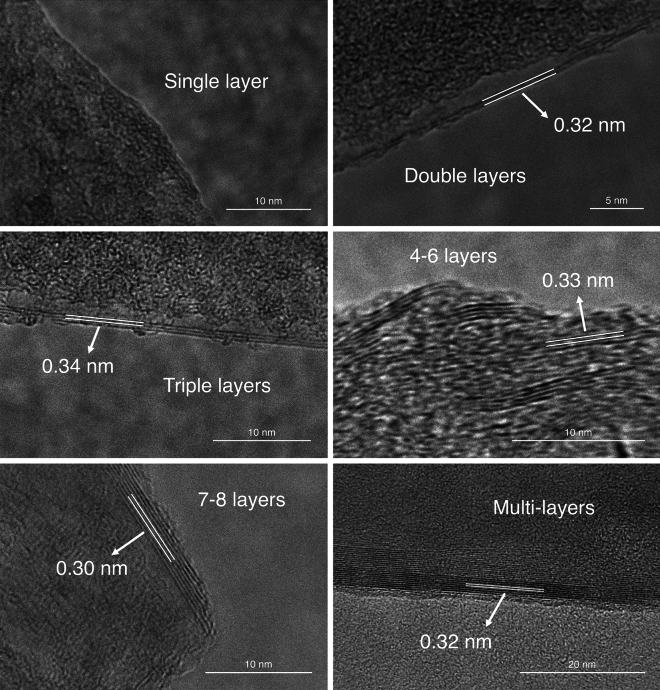
HRTEM images of the 5/5 Pencil Lead, containing various number of layers, ranging from single layer, few layers and multi-layer.

Figure 4 presents the high resolution TEM (HRTEM) images of 5/5 exfoliated pencil lead at different areas and confirmed that single layer can be exfoliated from our methods. The exfoliated graphene product presented various number of layers graphene, from single to multi-layer, which can also be observed from all other samples. These HRTEM images confirmed that our method results in a mixed product of single layer and multiple layers graphene, demonstrating the potential for single layer graphene application as well. The characteristic interlayer spacing of graphene, approximately around 0.3 nm, is also shown in Fig. 4.

All characterization methods collectively confirm that the exfoliated product comprises single-layer graphene mixed with graphite flakes from both pencil lead and HOPG. The graphene obtained is remarkably pure and does not exhibit characteristics typical of graphene oxide. The quality of graphene exfoliated from HOPG, and pencil lead is comparable, yet they possess distinct characteristics. Graphene exfoliated from pencil lead exhibits a smaller and more rigid structure, while HOPG graphene is fabric-like and features wrinkles. As a result, these two products potentially serve different applications.

### Graphene from Pencil leads vs HOPG deposited on electrodes

**Figure 5:**
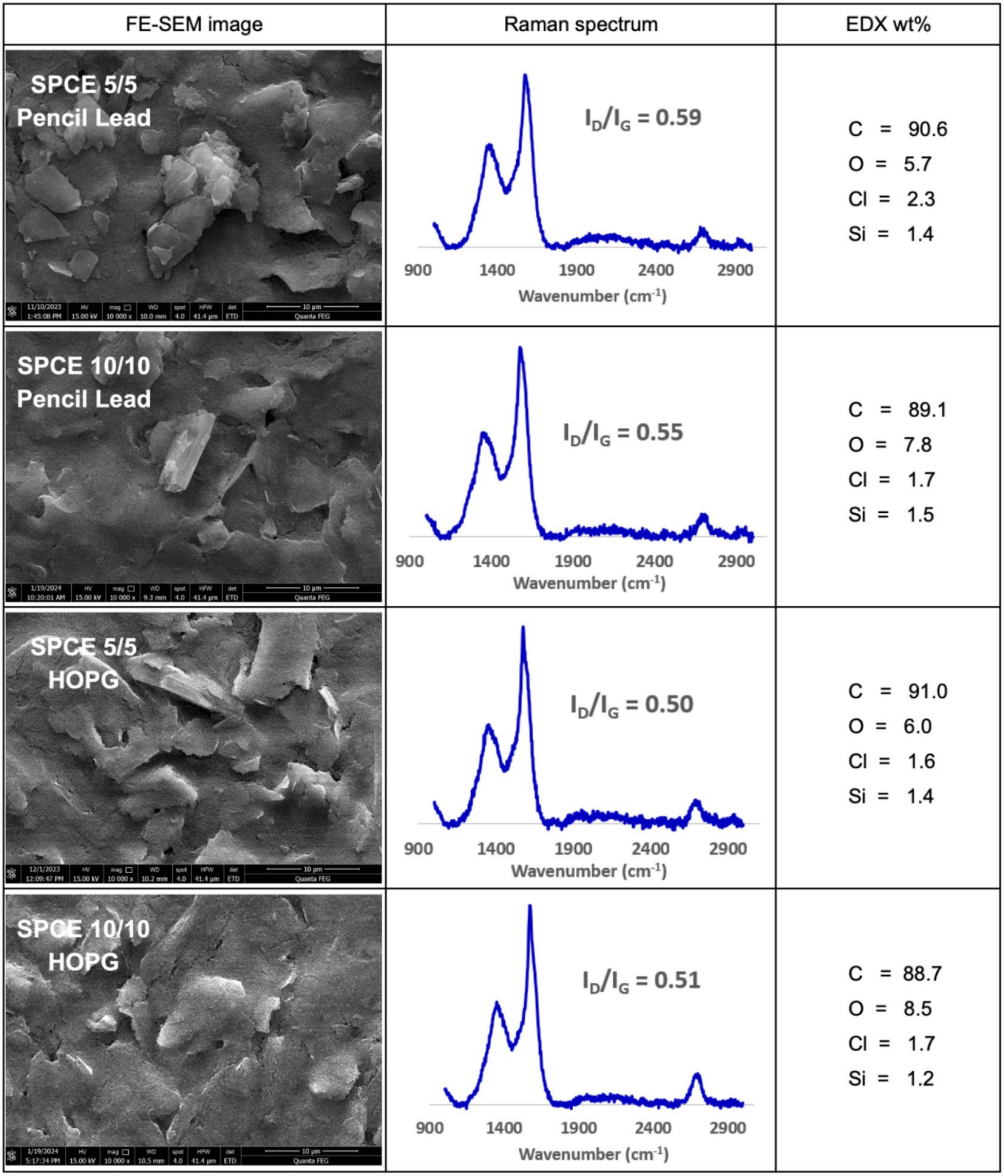
FESEM images, Raman spectra, and EDS weight percentage of: 5/5 Pencil Lead on SPCE, 10/10 Pencil Lead on SPCE, 5/5 HOPG on SPCE, and 10/10 HOPG on SPCE.

FESEM images of SPCE deposited 5/5 Pencil Lead, 10/10 Pencil Lead, 5/5 HOPG and 10/10 HOPG are shown in Fig. 5. From the images, graphene flakes can be clearly observed on all four deposited SPCE. The 5/5 conditions resulted in a higher uniformity and more continuous film of graphene deposition than the 10/10 conditions, regardless of carbon sources used, which aligns with the film thickness values obtained from AFM characterization. When comparing the Pencil Lead conditions to their HOPG counterparts, the graphene layers from pencil lead are more rigid flakes with a smaller particle size, whereas HOPG presents larger, thin layers of graphene. This aligns with the characteristics we observed for powdered graphene products after exfoliation in the earlier results section. Regarding the EDS spectra, an increment in the percentage of carbon can be realized for the deposited electrodes compared to bare SPCE. Consistent with the FESEM results, the amount of carbon is comparable between Pencil Lead conditions and the HOPG conditions. The amount of oxygen observed also matches that of carbon, with a lower amount of oxygen observed in the 5/5 conditions than in the 10/10 conditions. The 10/10 sample, having twice the duration of applied potential during exfoliation process compared to the 5/5 sample, may elevate the risk of oxidation in the graphene product consequently leading to a higher percentage of oxygen content. The presence of Cl is hypothesized to be the result of the NaCl used in the deposition process. Its occurrence in the bare SPCE suggests that Cl as well as silicon are contaminants originating from the electrode.

The results of the graphene conditions deposited on SPGE, as depicted in Supplementary Fig. S4, provide evidence of successful graphene deposition similar to SPCE. However, a smaller amount of deposition is observed in both conditions for HOPG graphene compared to the Pencil Lead products. Thick and rigid graphene flakes are evident in both Pencil Lead conditions, contrasting with the curvy sheet-like structure of HOPG layers. The EDS results shown in Supplementary Fig. S4 reveal that the 5/5 Pencil Lead sample exhibited the highest carbon content with no detectable oxygen. This is hypothesized to be a result of a shorter duration between the synthesis and characterization processes. Despite this, the ability to achieve such a low amount of oxygen during deposition is remarkable. Similar to the results from SPCE, the presence of Na is likely from the NaCl solution used for deposition, while gold is attributed to the sputtering process. Overall, the morphology and elemental composition analyses resemble those of graphene modified on SPCE.

Analysis of the Raman spectra of the SPCEs and SPGEs indicates the successful deposition of graphene onto the electrodes, as evidenced by the distinct peaks at 1350, 1580, and 2685 cm^−1^, which correspond to the D, G, and 2D peaks of graphene, respectively. An increased I_D_/I_G_ and a decrease in the intensity of the 2D peaks compared to those of graphene powder, implying that an increase in defects and graphene layers has occurred during the deposition process, especially in SPCE. Additionally, both SPCE 5/5 and 10/10 Pencil Lead exhibit comparable I_D_/I_G_ values, with respective ratios of 0.59 and 0.55, when compared to their HOPG counterparts at 0.50 and 0.51. Similarly, the I_D_/I_G_ ratios of SPGE 5/5 and 10/10 Pencil Lead are in line with to their HOPG counterparts, indicating a comparable level of defects.

Although there is a slight deposition difference observed from the FESEM images, Pencil Leads conditions displayed more pronounced deposition than their HOPG counterparts and the overall structure or morphology is quite similar for both SPCE and SPGE after deposition. This is evident from the relatively small amount of graphene deposited on the surface as compared to the structure obtained from the graphene synthesis process. The Raman spectra and the atomic percentages obtained from EDS showed mixed results, with 5/5 Pencil Lead having the higher carbon content while showing a higher number of defects from the Raman spectra. However, the disparities are negligible when compared to each respective value. Therefore, it could be concluded that the high-quality graphene, obtained through our exfoliation process using either pencil lead and HOPG, can be deposited onto electrodes, demonstrating comparable physical characteristics, including minimal defects and high carbon content.

### Comparative analysis of graphene from pencil lead deposited on SPCE vs SPGE

**Figure 6:**
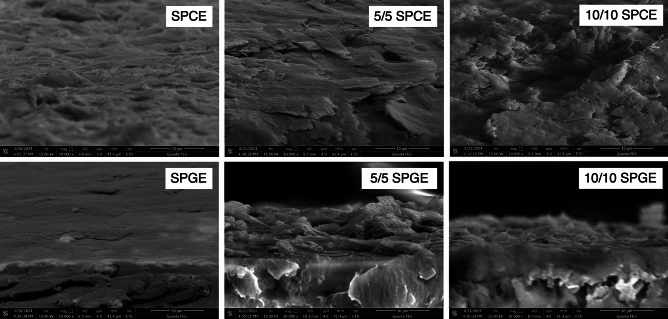
FESEM images (cross-sectional view) of Bare SPCE, Bare SPGE, 5/5 Pencil Lead on SPCE, 10/10 Pencil Lead on SPCE, 5/5 Pencil Lead on SPGE, 10/10 Pencil Lead on SPGE.

The comprehensive examination on the physical properties and structural analysis of graphene from pencil lead deposited on SPCE and SPGE were analyzed with XPS, FTIR and FESEM. Firstly, the magnified view of cross-sectional images of graphene deposited on SPCE and SPGE were compared with the bare electrodes in Fig. 6. All 5/5 and 10/10 exfoliated graphene layers covered throughout the surface on SPCE and SPGE. Smoother and satisfactorily deposited graphene layers were observed on SPCE electrode, whereas coarser and discontinuous graphene formed on SPGE electrodes. Comparing between exfoliated conditions, 10/10 exfoliated graphene exhibited smaller particles; however, larger particles were presented in 5/5 graphene. The results align with the size analysis of exfoliated graphene powder conducted earlier for both SPCE and SPGE as the longer the exfoliation time damage graphene particles. The full images with various magnification can be found in Supplementary Fig. S5.

**Figure 7:**
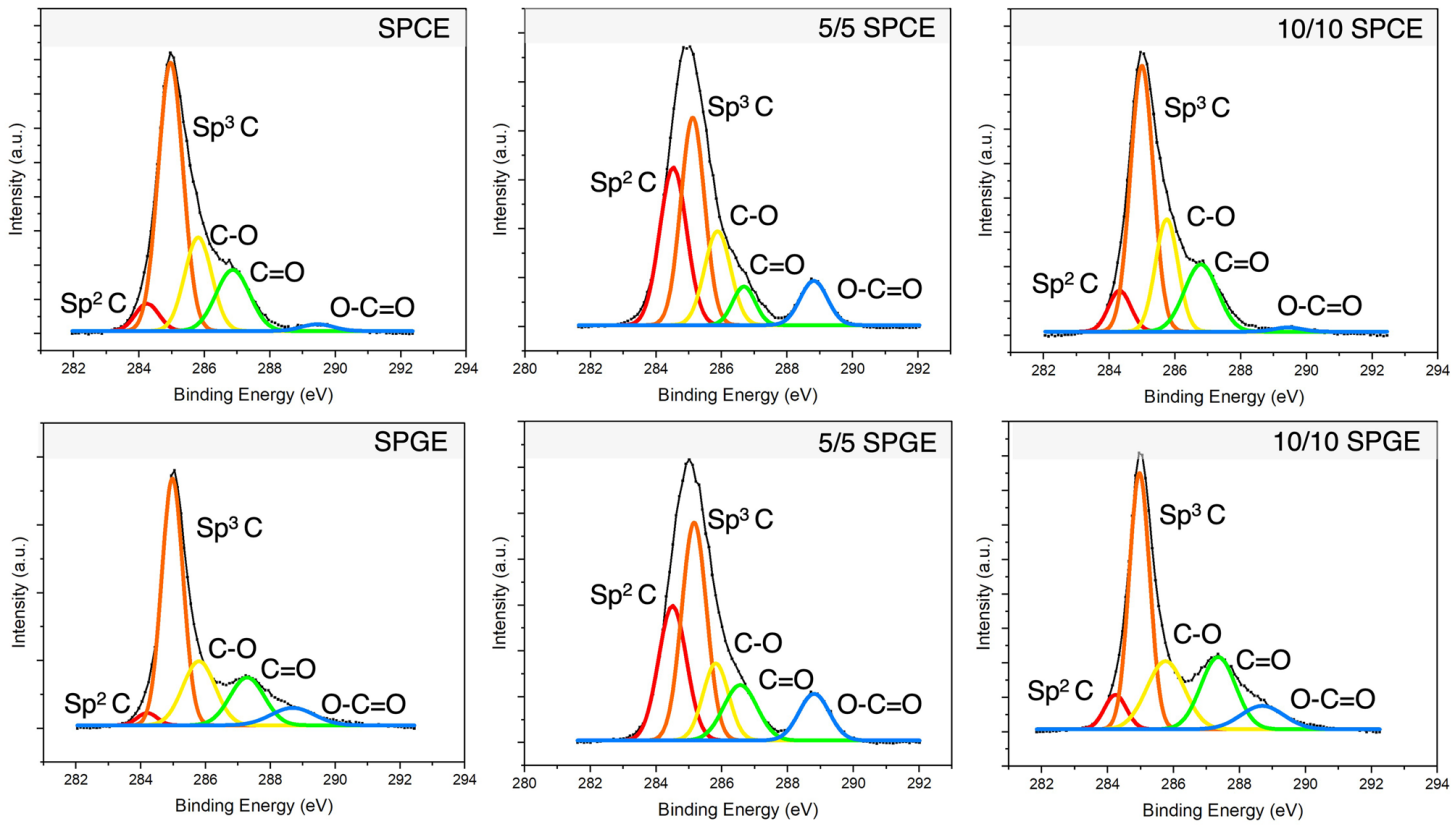
C 1s high resolution XPS spectra and peaks deconvolution of Bare SPCE, Bare SPGE, 5/5 Pencil Lead on SPCE, 10/10 Pencil Lead on SPCE, 5/5 Pencil Lead on SPGE, 10/10 Pencil Lead on SPGE. XPS spectral peak deconvolutions were performed using OriginLab version 2024b.

The X-ray photoelectron (XPS) spectra of high-resolution C 1s peak of graphene modified and unmodified on SPCE and SPGE were presented with their deconvolution peaks of carbon bonding in Fig. 7. All samples presented characteristic peak of carbon binding energy where sp^2^ carbon, sp^3^ carbon, C–O, C=O, and O–C=O bonding correspond to binding energy of 284.5, 285, 286, 288 and 289 eV, respectively^19^. The result of curve fitting for each sample were presented with peak positions (binding energy), percent area after deconvolution, and coefficient of determination (R^2^) of curves fitting (Supplementary Table S2). It was confirmed that in all samples, the graphene-modified samples present higher sp^2^ carbon peak at around 284–284.5 eV binding energy in compare with bare SPCE and SPGE. Especially, the 5/5 graphene exfoliated from pencil lead demonstrated high percent area of sp^2^ peak as 31% and 26% on SPCE and SPGE electrodes respectively. While 10/10 presents a lower portion of sp^2^ than in 5/5 condition, it is still better than their respective bare electrodes. The C–O bonding potentially corresponding to epoxide (C–O–C) or hydroxylated carbon (C–OH) are quite similar in all samples which mainly consider planar defect where 5/5 exfoliated graphene showed small value compare with their bare and 10/10 samples. The two peaks that were inversely proportional to each other are C=O and O–C=O represented the edge defect of graphene. From the result, when one peak is high, the other will be low, and vice versa. Overall, the combination of defect peaks compares with carbon peaks (sp^2^ C and sp^3^ C), 5/5 deposited sample showed better quality of graphene layer deposited on electrodes with high percentage of carbon. Comparing between the different kind of electrodes, graphene deposit on the SPCE presented less surface defect than deposited on SPGE which agree well with FESEM cross-section images. The exfoliated graphene deposited on SPCE is better than that on SPGE, with 5/5 deposited on SPCE demonstrated the best condition. In addition, it was supported by FTIR results that C–OH defect was abundantly presented on both unmodified SPCE and SPGE which exhibited the O–H stretching at around 3500 cm^−1^ in FTIR spectra (Supplementary Fig. S6). On the other hand, the C–O bonding of deposited graphene samples could correspond to epoxide. In this case, the graphene-modified samples are more chemically stable than unmodified electrodes. Other peaks of the FTIR were similar in all samples, possibly due to the penetration depth which is around several microns being much deeper than the deposition thickness. The surface information of our graphene film was barely acquired to characterize the graphene film, which had a thickness below several hundred nanometers. Most of the information from FTIR analysis could be overwhelmed by the electrode layers.

### Electrochemical measurement of exfoliated graphene on SPCE vs SPGE

**Figure 8:**
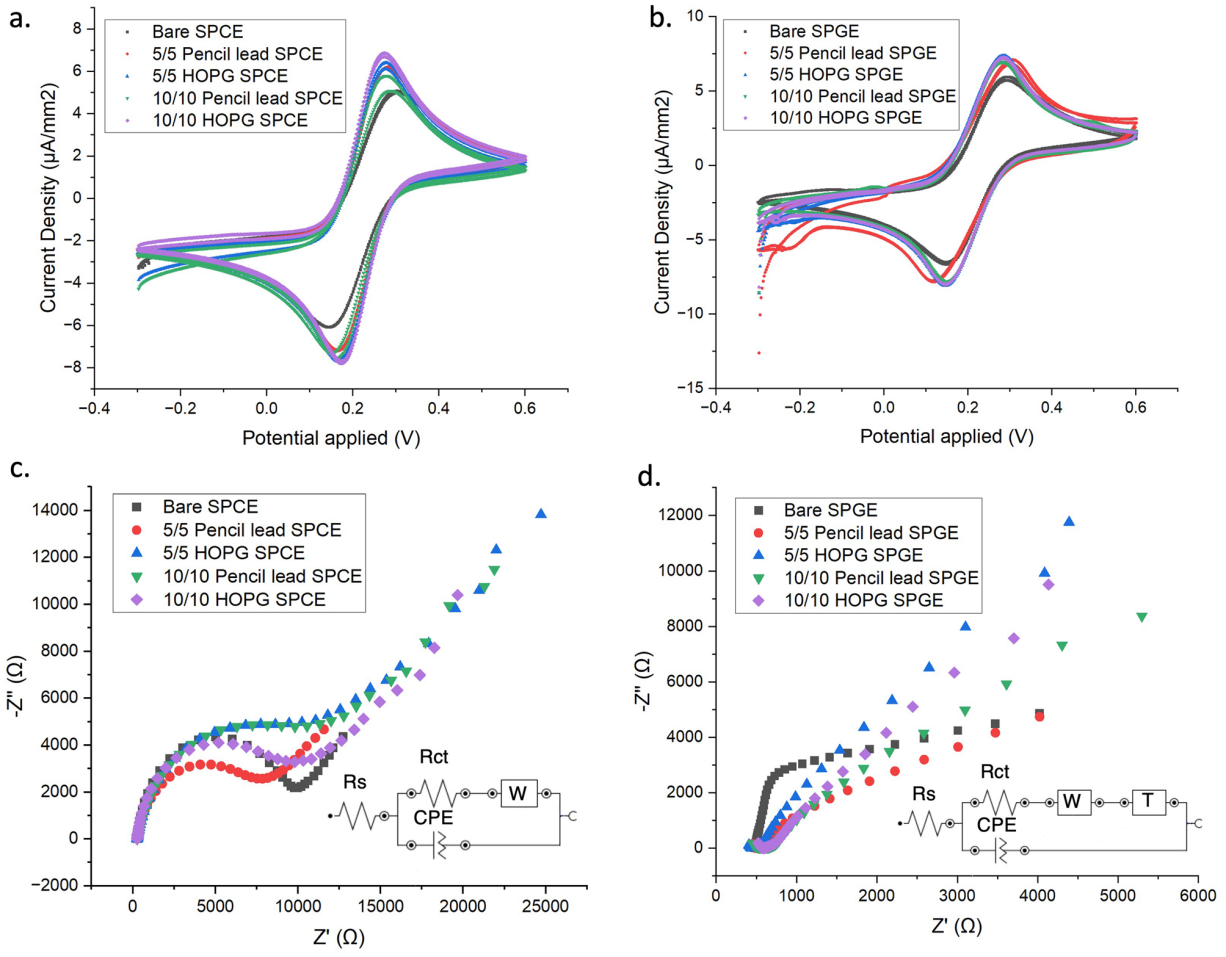
Cyclic voltammograms of unmodified and modified exfoliated graphene (5/5 Pencil Lead, 10/10 Pencil Lead, 5/5 HOPG, and 10/10 HOPG) on (**a**) SPCE and (**b**) SPGE. EIS measurements of unmodified and modified exfoliated graphene (5/5 Pencil Lead, 10/10 Pencil Lead, 5/5 HOPG, and 10/10 HOPG) on (**c**) SPCE and (**d**) SPGE. All CV and EIS plots were created by using OriginLab version 2024b.

Utilizing cyclic voltammetry allows for a comparison of the redox characteristics between SPCE and SPGE electrodes and their graphene modifications; both 5/5 and 10/10 graphene exfoliated from HOPG and pencil lead, as depicted in Fig. 8a, b. The cyclic voltammogram (CV) reveals a similar pattern in the characteristic redox behavior of the ferrocyanide/ferricyanide, with SPGE exhibiting lower redox potential couples and higher current peaks than SPCE electrodes, indicating greater sensitivity and overall higher conductivity. The current density peak of bare and modified SPGE is found to be higher than bare SPCE in all cases. This indicates that SPGE has a better electrochemical conductivity overall. Our functionalized exfoliated graphene on both electrodes presented higher current peaks for all cases compared with their bare electrodes. Thus, the graphene modification exhibited the enhancement in electrochemical properties on both SPCE and SPGE.

The EIS graphs of SPCE and SPGE with graphene deposited samples under various conditions, along with their respective equivalent circuits were depicted in Fig. 8c, d. Simple Randle model is common for electrolytic reaction of solution analytes. The model consists of solution resistance (R_s_) in series with parallel combination between electric double layer capacitance (C_dl_) and charge transfer resistance (R_ct_). The constant phase element (CPE) was implemented in place of electrical double layer capacitance to define the non-ideal capacitance properties of SPCE and SPGE constructed with carbon-base ink material with other components. The simple circuit was modified with the Warburg impedance (W) in all cases to represent diffusion during charge transfer process (bottom inset of Fig. 8c). The simple Randle circuit with Warburg diffusion can fit all EIS results except for graphene deposited on SPGE samples. Both 5/5 and 10/10 graphene deposited on SPGE exhibited distinct EIS curve, which could not be well fitted with the earlier model. The tails of Nyquist plot were tangent with different angle apart from 45°. In this case, the Tangent hyperbolic diffusion (T), accounted for the film covered with electroactive species or porous film, can be applied to represent this characteristic curve where diffusions occurred separately on the electrode and electroactive species, in this instant, exfoliated graphene. The graphene deposited on SPGE displayed the discontinuity on the deposition area evident in FESEM images (Fig. 6). The electrolyte could interact with both surface of SPGE and graphene layers. As a result, the model circuit for graphene deposited on SPGE samples were applied the Tangent hyperbolic diffusion (T) in series with Warburg impedance (W) to represent diffusion process on separated surfaces as depicted in the bottom inset of Fig. 8d.

The charge transfer resistance obtained from fitting the model circuits with EIS curves to facilitate a comparison of the electrical properties of the graphene-modified electrodes, as presented in the bar graph (Supplementary Fig. S7). Lower R_ct_ indicates the higher conductivity of the sample. With the deposited graphene, the R_ct_ decreased in all cases compared to bare SPCE and SPGE. The results were in agreement with CV results earlier. For comparison of the exfoliated conditions, most of the 5/5 exfoliated graphene samples demonstrated lower resistance compared to their 10/10 counterpart. The 5/5 samples, with a shorter exfoliation time, may result in less damage to the exfoliated graphene product, leading to improved electrical properties. The EIS results were in consistent with previous physical and chemical characterization in earlier sections. Our graphene product derived from pencil lead proved to enhance the electrical performance of both SPCE and SPGE and comparable with the costly HOPG graphene samples which leads to many useful applications.

## Methods

### Graphene electrochemical exfoliation

Commercial 0.5 mm 4B pencil lead (Pentel) purchased from a local stationery store and Highly Oriented Pyrolytic Graphite (HOPG) purchased from Sigma-Aldrich were used as raw materials for the comparative study of electrochemical graphene exfoliation. The electrolyte solution was prepared by combining 100 mL of 0.49 M H_2_SO_4_ and 11 mL of 30% KOH^20^. The electrochemical setup applied pencil lead as the cathode and a 99.99% platinum wire (Advent Research Materials) as the anode. Electrochemical exfoliation was performed using a constant potential of 2.5 V supplied by the potentiostat (Autolab PGSTAT204, Metrohm) for 60 s, followed by 10 cycles of alternating positive and negative voltage. Variations of the time used for the cycles (alternating positive/negative voltage) are 2/2, 5/5, and 10/10 s. The electrolyte solution with the graphene is then filtered using vacuum filtration. The graphene was further intercalated with 20 mL of 99.5% DMF and sonicated for 5 min at room temperature. Finally, the sonicated solution is heated at 80 °C using the hot plate to evaporate the DMF residue.

### Deposition of graphene on electrodes

The dried synthesized graphene is combined with 10 mL of 0.8766 M NaCl and sonicated for 5 min at room temperature. 200 μL of the solution is then dropped onto the screen-printed carbon electrode (SPCE) and screen-printed graphene electrode (SPGE) purchased from Quasense using Autolab Potentiostat. Two cycles of cyclic voltammetry staircase are then applied with a voltage ranging from −1.5 to 0.8 V and a scan rate of 0.02 V s^−1^^21^. After the deposition, the screen-printed electrode was rinsed with DI water and left to dry for 5 min. All deposition steps are repeated two times.

### Characterization

Both the synthesized graphene and the screen-printed electrodes with deposited graphene are characterized using various techniques. Quanta 250 FEG Field Emission Scanning Electron Microscope (FESEM) and Energy-dispersive X-ray spectroscopy (EDS) are used to observe the structure of graphene, its defects and elemental composition. JEOL JEM-2100 Transmission electron microscopy (TEM) is used to examine the inner structure of graphene and its diffraction pattern. Horiba XploRA PLUS Raman Spectroscopy is used to further analyze the structure of graphene such as its amount of defect and number of layers. X-ray photoelectron spectroscopy (XPS, PHI Versarprobe 4) is used for surface chemical analysis on the modified electrodes. The high-resolution C 1s peak analysis of XPS curves were fitted with Gaussian function using OriginLab (OriginPro, Version 2024b, https://www.originlab.com). Fourier Transform Infrared Spectrometer (FT-IR, Perkin Elmer, spectrum3) revealed the functional group of polar bonding vibrational mode.

### Size distribution analysis

The FESEM images were calibrated using the SEM scale bar and the pixel size was determined using ImageJ software in order to measure the pixel size and convert to µm. The area was measured by using the polygon selection feature to enclose the area of the graphene flakes. The data obtained from ImageJ (ImageJ, Version 1.54g, https://imagej.net/ij/download.html) was then exported to Python 3 (Python 3, Version 3.11.7, https://www.anaconda.com/download) for data visualization and statistical analysis.

### Stiffness test

The stiffness test of exfoliated graphene was done by preparing a 2 mm hole array on Silicon wafer using UV lithography and etching. First, a silicon (Si) master mold was fabricated using the conventional lithography and deep reactive ion etching (DRIE) process in cleanroom (CR) class 100. The surface of the 6-inch p-type Si(100) wafer was cleaned by standard cleaning (SC-1) process to remove unwanted contaminants. Then, a 1.0 μm-thick silicon dioxide (SiO_2_) was deposited onto the p-type Si(100) wafer by plasma-enhanced chemical vapor deposition (PECVD) method. Layers of 2.0 μm-thick photoresist (PR) with a 2.0 μm diameter hole pattern were fabricated by the conventional photolithography process. The PR pattern was transferred to the SiO_2_ layer by inductively coupled plasma-reactive ion etching (ICP-RIE) process (CF_4_/CHF_3_/Ar: 10/50/100 sccm, P: 150 mTorr, RF: 800 W/5 min). After the remaining PR film was stripped, 0.5 μm-thick AlSi (1%) Cu (0.5%) metal alloy film was deposited into the SiO_2_ trench using sputtering method (Gas pressure = 2.5 mTorr, RF power = 12 kWatt, at room temperature). Then, the exfoliated graphene powder was deposited on the hole arrays substrate for stiffness test. The AFM (JPK Instruments, Nanowizard3) was used to perform the nanoindentation by force spectroscopy measurements. The indentation was operated under two conditions which are extend and retract whereby the two data sets were combined to construct the force—distance curves.

### Electrochemical measurement

Graphene deposited on SPCE and SPGE is evaluated for electrical performance using Electrochemical Impedance Spectroscopy (EIS) and Cyclic Voltammetry (CV) with the Autolab potentiostat. Both methods are conducted in a 200 μL solution of 0.10 M KCl containing 5.0 mM K_3_Fe(CN)_6_. In the CV test, the voltage range is set from −0.3 to 0.6 V, and the scan rate is 0.05 V s^−1^. EIS analysis is carried out over a frequency range spanning from 0.2 Hz to 1 MHz, employing 60 frequencies and maintaining a fixed AC potential of 0.1 V.

## Conclusions

In summary, a simple and accessible method of graphene production involves a controlled electrochemical process, surfactant sonification, and filtering using pencil lead as the source material. The synthesized graphene is deposited onto SPCE and SPGE to analyze electrical performance. Various characterization methods, including FESEM, TEM, Raman spectroscopy, EDS, XPS, AFM, CV, and EIS, revealed that the exfoliated graphene from pencil lead exhibits properties comparable to commercially available graphene. Additionally, the graphene exfoliated from pencil lead and deposited on electrodes demonstrates superior conductivity and electrical performance compared to bare electrodes. This finding raises a simple and cost-effective method producing high-quality graphene, paving the way for large-scale graphene production, and advancing future sensor technologies.

### Supplementary Information

Supplementary Information.

## Data Availability

Data is provided within the manuscript or supplementary information files.

## References

[1] Yang G, Li L, Lee WB, Ng MC (2018). Structure of graphene and its disorders: A review. Sci. Technol. Adv. Mater..

[2] Novoselov KS (2004). Electric field effect in atomically thin carbon films. Science.

[3] Avouris P, Dimitrakopoulos C (2012). Graphene: Synthesis and applications. Mater. Today.

[4] Yi M, Shen Z (2015). A review on mechanical exfoliation for the scalable production of graphene. J. Mater. Chem. A.

[5] Hummers WS, Offeman RE (1958). Preparation of graphitic oxide. J. Am. Chem. Soc..

[6] Parvez K, Yang S, Feng X, Müllen K (2015). Exfoliation of graphene via wet chemical routes. Synth. Metals.

[7] Muñoz R, Gómez-Aleixandre C (2013). Review of CVD synthesis of graphene. Chem. Vapor Depos..

[8] Tan H, Wang D, Guo Y (2018). Thermal growth of graphene: A review. Coatings.

[9] Parvez K (2014). Exfoliation of graphite into graphene in aqueous solutions of inorganic salts. J. Am. Chem. Soc..

[10] Whitener KE, Sheehan PE (2014). Graphene synthesis. Diamond Relat. Mater..

[11] Abdelkader AM, Cooper AJ, Dryfe RAW, Kinloch IA (2015). How to get between the sheets: A review of recent works on the electrochemical exfoliation of graphene materials from bulk graphite. Nanoscale.

[12] Alabdo F (2023). An environmentally friendly and simple method for producing multi-layer exfoliated graphene in mass production from pencil graphite and its utilization for removing cadmium from an aqueous medium. Carbon Lett..

[13] Anwar MA (2019). Electrochemical exfoliation of pencil graphite core by salt electrolyte. IOP Conf. Ser.: Mater. Sci Eng..

[14] Chen K, Xue D, Komarneni S (2017). Nanoclay assisted electrochemical exfoliation of pencil core to high conductive graphene thin-film electrode. J. Colloid Interface Sci..

[15] Loudiki A (2022). Preparation of graphene samples via electrochemical exfoliation of pencil electrode: Physico-electrochemical characterization. Appl. Surface Sci. Adv..

[16] Singh VV (2012). Greener electrochemical synthesis of high quality graphene nanosheets directly from pencil and its SPR sensing application. Adv. Funct. Mater..

[17] Zandiatashbar A (2014). Effect of defects on the intrinsic strength and stiffness of graphene. Nat. Commun..

[18] Hadi A, Zahirifar J, Karimi-Sabet J, Dastbaz A (2018). Graphene nanosheets preparation using magnetic nanoparticle assisted liquid phase exfoliation of graphite: The coupled effect of ultrasound and wedging nanoparticles. Ultrason. Sonochem..

[19] Shchukarev A, Korolkov D (2004). XPS study of group IA carbonates. Central Eur. J. Chem. CENT EUR J CHEM.

[20] Su C-Y (2011). High-quality thin graphene films from fast electrochemical exfoliation. ACS Nano.

[21] Alonso RM, San-Martín MI, Sotres A, Escapa A (2017). Graphene oxide electrodeposited electrode enhances start-up and selective enrichment of exoelectrogens in bioelectrochemical systems. Sci. Rep..

