# Supplementary material for: Electrochemical exfoliation of graphene from pencil lead
Source: Sci Rep. 2024 Jul 10;14:15892. doi: 10.1038/s41598-024-66825-0 (PMC11236967; doi:10.1038/s41598-024-66825-0)
Supplement: Supplementary file 1 — Supplementary Information. [file 41598_2024_66825_MOESM1_ESM.pdf]

Supplementary Information

Electrochemical Exfoliation of Graphene from Pencil Lead

Natchanon Jiwarawat[1], Thapan Leukulwatanachai[1], Kunbhass Subhakornphichan[1], Siwagorn Limwathanagura[1], Sittinadh Wanotayan[2] , Nithi Atthi[3], Apirak Pankiew[3] and Porpin Pungetmongkol[1,*]

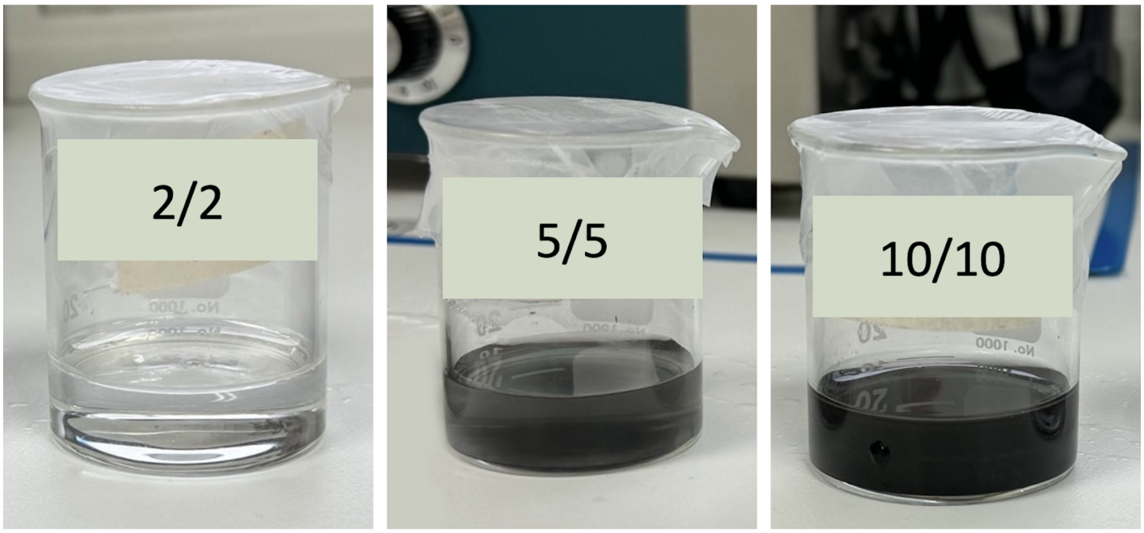

Figure S1. The graphene products after synthesis in 3 conditions with different exfoliation periods (2, 5, and 10 seconds) for each alternating potential (2/2, 5/5, and 10/10).

Table S1. EDS of sources material (HOPG and Pencil leads) compared with EDS of graphene powder exfoliated from each source with 2 conditions (5/5 and 10/10 exfoliations).

Sample	C wt%	O wt%	S wt%	K wt%	P wt%	Sn wt%	Si wt%	Cl wt%
HOPG	94.3	5.7	0	0	0	0	0	0
Pencil Lead	88.9	5.4	0	0	2.3	1.8	1.66	0
5/5 Pencil Lead Powder	60.4	34.1	2.9	1.7	0	0	0.8	0
10/10 Pencil Lead Powder	58	34.5	3.6	2.9	0	0	1	0
5/5 HOPG Powder	68.4	19.6	5.4	6.6	0	0	0	0
10/10 HOPG Powder	70.2	26	3	0.8	0	0	0	0

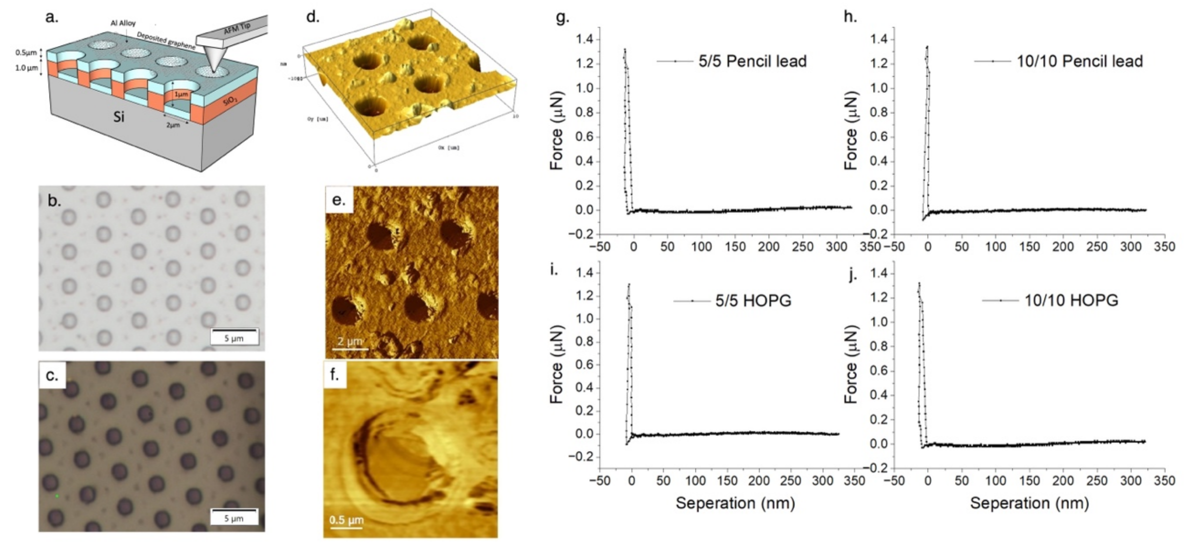

Figure S2. Stiffness analysis of sample. (**a**) Schematic of AFM nanoindentation for mechanical analysis, (**b**) Optical microscope image of micro holes array fabricated on Silicon wafer for the stress test before, and (**c**) after deposited with graphene, (**d**) 3D image from AFM scan area of micro holes array (**e**) Top view of AFM image, (**f**) Magnified view of graphene deposited on hole for mechanical test, and Force-separation curves of (**g**) 5/5 Pencil Lead, (**h**) 10/10 Pencil Lead, (**i**) 5/5 HOPG, and (**j**) 10/10 HOPG. Force-distance curves were generated, and the slopes were obtained using OriginLab software version 2024b.

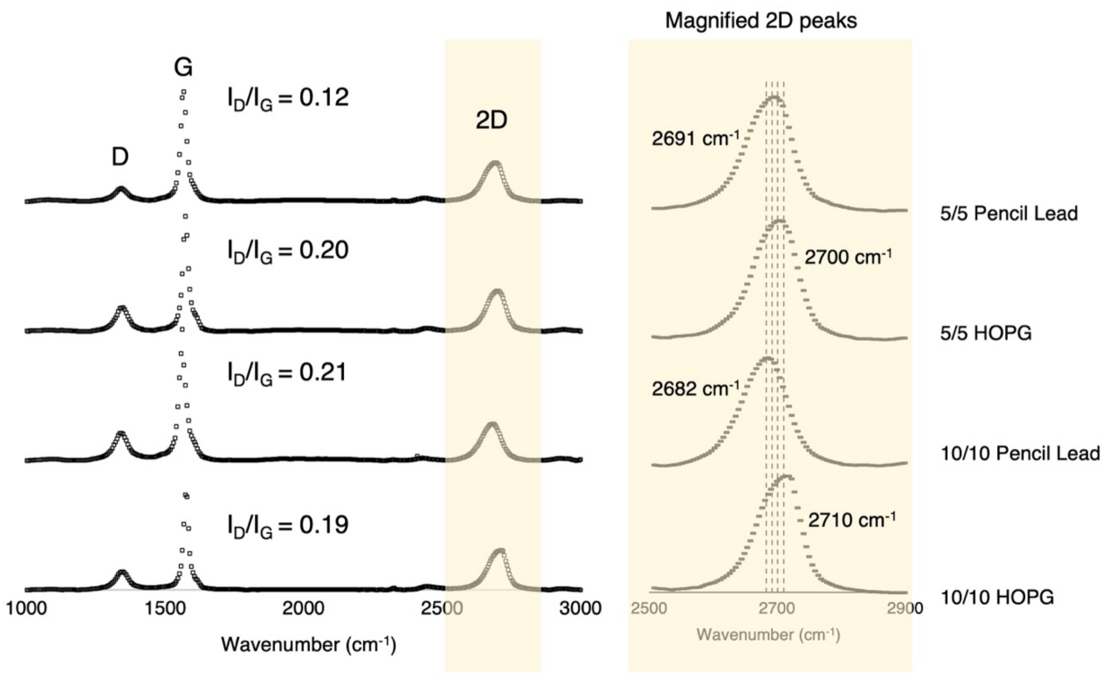

Figure S3. (left) Raman spectra of 5/5 Pencil Lead, 10/10 Pencil Lead, 5/5 HOPG, and 10/10 HOPG, showing I_D/I_G ratio. (right) Magnified Raman spectra showing 2D peaks of 5/5 Pencil Lead, 10/10 Pencil Lead, 5/5 HOPG, and 10/10 HOPG.

FE-SEM image	Raman spectrum	EDX wt%
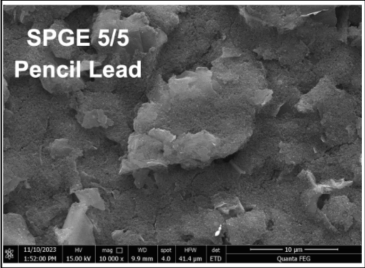	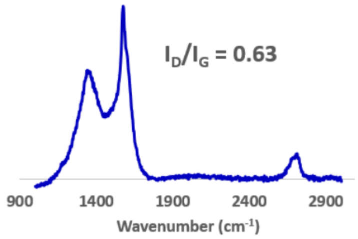	C = 95.7<br>Au = 4.3
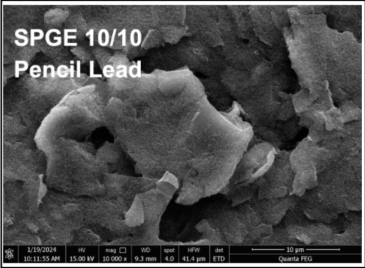	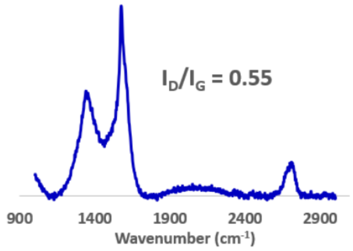	C = 90.3<br>O = 9.7
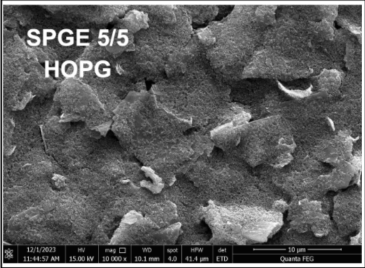	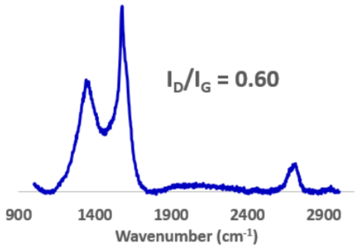	C = 84.6<br>Au = 8.9<br>O = 5.7<br>Na = 0.8
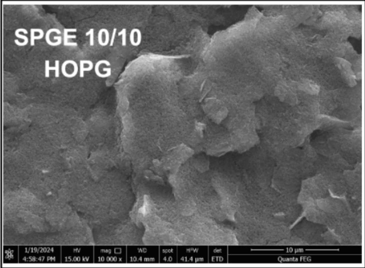	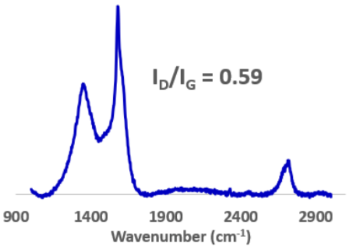	C = 89.8<br>O = 9.0<br>Na = 1.2

Figure S4. FE-SEM images, Raman spectra, and EDX weight percentage of: 5/5 Pencil Lead on SPGE , 10/10 Pencil Lead on SPGE , 5/5 HOPG on SPGE , and 10/10 HOPG on SPGE.

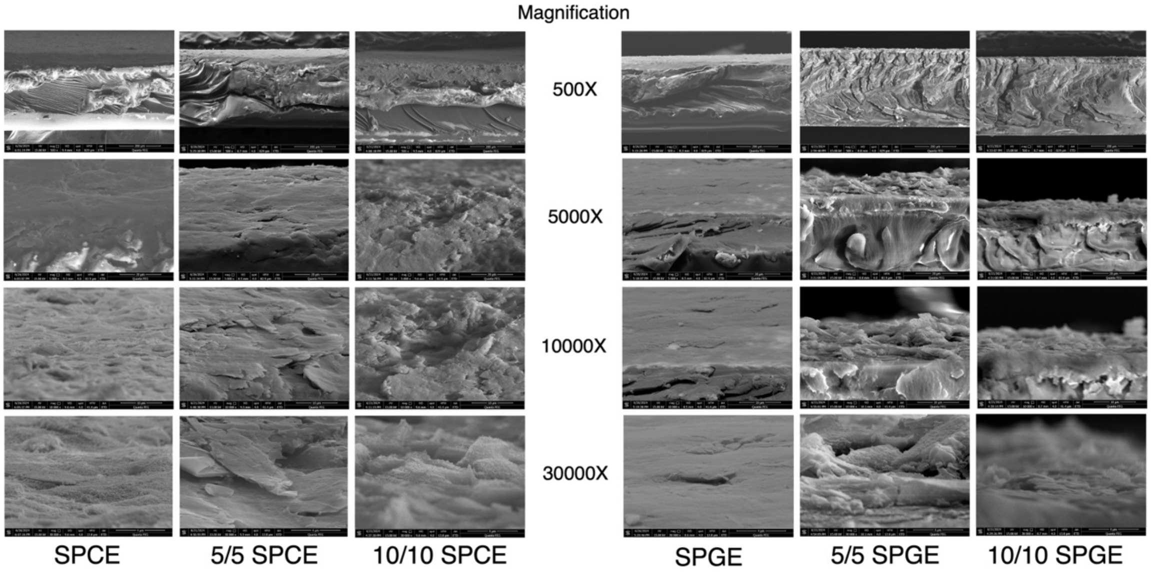

Figure S5. FE-SEM images (cross-sectional view) of: Bare SPCE, 5/5 Pencil Lead on SPCE, 10/10 Pencil Lead on SPCE, Bare SPGE, 5/5 Pencil Lead on SPGE, and 10/10 Pencil Lead on SPGE at different magnifications.

Table S2. The binding energy (eV), percent area, combination of carbon peaks, and combination of defect peaks, and R^2 of fitted peaks for C1s high resolution XPS spectra of samples: Bare SPCE, 5/5 Pencil Lead on SPCE, 10/10 Pencil Lead on SPCE, Bare SPGE, 5/5 Pencil Lead on SPGE, 10/10 Pencil Lead on SPGE. All data points were obtained from a Gaussian fit simulation to deconvolute XPS spectra using OriginLab software version 2024b.

Sample	SPCE		5/5 SPCE		10/10 SPCE		SPGE		5/5 SPGE		10/10 SPGE	
Peak	Binding Energy (eV)	(Area %)	Binding Energy (eV)	(Area %)	Binding Energy (eV)	(Area %)	Binding Energy (eV)	(Area %)	Binding Energy (eV)	(Area %)	Binding Energy (eV)	(Area %)
sp2	284.25	(5.35)	284.52	(31.02)	284.31	(7.69)	284.20	(2.47)	284.50	(26.09)	284.23	(6.27)
sp3	284.96	(54.94)	285.12	(35.95)	284.99	(51.06)	284.97	(52.77)	285.16	(37.48)	284.96	(43.51)
C-O	285.81	(20.93)	285.88	(17.91)	285.75	(21.39)	285.80	(20.78)	285.81	(13.67)	285.75	(21.13)
C=O	286.86	(16.98)	286.68	(6.33)	286.79	(18.89)	287.28	(16.46)	286.56	(12.93)	287.35	(20.77)
O-C=O	289.45	(1.80)	288.81	(8.79)	289.43	(0.97)	288.68	(7.52)	288.80	(9.83)	288.69	(8.32)
Sum carbon peaks (Area %)	60.29%		66.97%		58.75%		55.24%		63.57%		49.77%	
Sum defect peaks (Area %)	39.71%		33.03%		41.25%		44.76%		36.43		50.23%	
R^2	0.9977		0.9975		0.9975		0.9939		0.9966		0.9948	

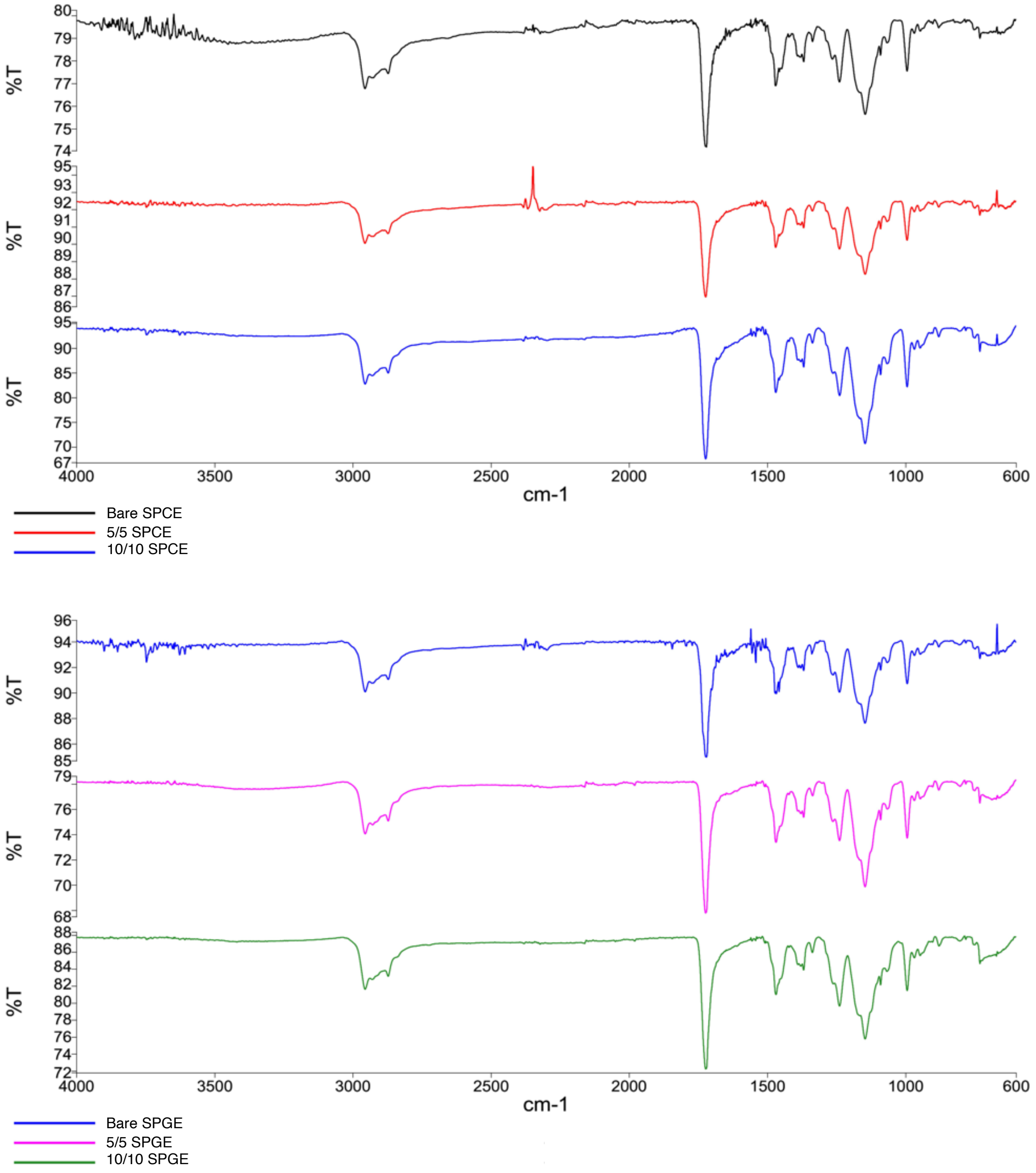

Figure S6. (top) FTIR spectra of Bare SPCE, 5/5 Pencil Lead on SPCE, and 10/10 Pencil Lead on SPCE. (bottom) Bare SPGE, 5/5 Pencil Lead on SPGE, and 10/10 Pencil Lead on SPGE.

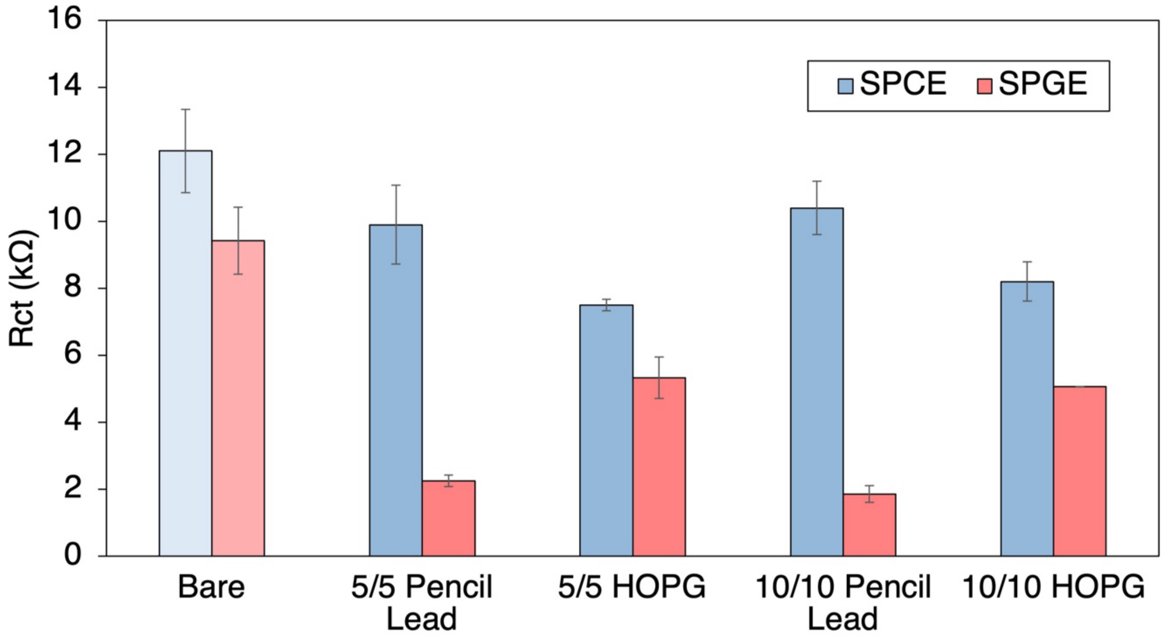

Figure S7. Charge transfer resistance (R_{ct}) of Bare SPCE, Bare SPGE, and 5/5 Pencil Lead, 5/5 HOPG, 10/10 Pencil Lead, 10/10 HOPG deposited on SPCE and SPGE.
